# A Strategy for Tuning Electron–Phonon Coupling and Carrier Cooling in Lead Halide Perovskite Nanocrystals

**DOI:** 10.3390/nano13243134

**Published:** 2023-12-13

**Authors:** Huafeng Shi, Xiaoli Zhang, Ruxue Li, Xinhai Zhang

**Affiliations:** 1 Department of Electrical and Electronic Engineering, Southern University of Science and Technology, Shenzhen 518055, China; 2 Center of Attosecond Science, Songshan Lake Materials Laboratory (SLAB), Dongguan 523808, China; 3 Guangdong Provincial Key Laboratory of Information Photonics Technology, School of Physics and Opto–Electronic Engineering, Guangdong University of Technology, Guangzhou 510006, China

**Keywords:** electron–phonon coupling, carrier cooling, perovskites, light–induced lattice expansion

## Abstract

Perovskites have been recognized as a class of promising materials for optoelectronic devices. We intentionally include excessive Cs^+^ cations in precursors in the synthesis of perovskite CsPbBr_3_ nanocrystals and investigate how the Cs^+^ cations influence the lattice strain in these perovskite nanocrystals. Upon light illumination, the lattice strain due to the addition of alkali metal Cs^+^ cations can be compensated by light–induced lattice expansion. When the Cs^+^ cation in precursors is about 10% excessive, the electron–phonon coupling strength can be reduced by about 70%, and the carrier cooling can be slowed down about 3.5 times in lead halide perovskite CsPbBr_3_ nanocrystals. This work reveals a new understanding of the role of Cs^+^ cations, which take the A–site in ABX_3_ perovskite and provide a new way to improve the performance of perovskites and their practical devices further.

## 1. Introduction

Perovskites have been recognized as a class of promising materials for optoelectronic devices, including photovoltaic cells [1], light–emitting diodes [2], photodetectors [3], and other devices. Especially, it is a positive attempt to surpass the Shockley–Queisser efficiency limitation for solar cells by means of metallization of perovskite [4]. In a typical ABX_3_ structure, the A–site is the monovalent organic or inorganic cation, the B–site is the divalent metal cation, and the X–site is the halogen anion. The halogen anion at the X–site is an important factor affecting the bandgap of a perovskite. The emission of perovskites covers the visible spectral range through the ion exchange of the halogen anion at the X–site [5]. The halogen anion at the X–site also plays an important role in exciton in perovskites, resulting in intense white–light emission [6]. The divalent metal cation at the B–site determines the framework of the steric structure and the electronic structure of a perovskite [7]. The environment–friendly perovskites are also achieved by the substitution of the metal cation at the B–site [8]. The metal cations at B–sites act as localized charge traps on the nanocrystal surface, reducing the charge extraction rate of perovskite devices [9]. Mixing organic and inorganic cations at the A–sites is effective in adjusting the stacking of atoms, thus decreasing the distortion of the lattice and boosting the efficiency and stability of perovskites [10]. It can inhibit the formation of the “yellow phase” and improve the light stability of perovskites [11].

It has also been found that alkali metal elements have versatile and profound effects, such as improving the homogeneity of perovskite films [12], decreasing the defect density in perovskites [13], and slowing the hot–carrier cooling in perovskites [14]. Even very low concentrations of alkali metal cations added into precursors can greatly affect the crystallization kinetics of perovskites and significantly reduce defect density to boost the efficiency of perovskite devices [15]. In the alkali metal elements, the rubidium cation (Rb^+^) has too small a radius to occupy the A–site of the perovskites [16] but increases the mobility of the carriers in perovskites [17]. The potassium cation (K^+^) decorates the grain boundaries and surface of the nanocrystals, substantially reducing photo–induced ion migration in perovskites [18]. Potassium chloride (KCl) suppresses the interfacial carrier recombination in the perovskite solar cells by interfacial modification [19]. The addition of sodium cations (Na^+^) remarkably improves the quality of perovskites, including enlarging the grain sizes and reducing the density of defects, thus increasing the power conversion efficiency of perovskite solar cells [20]. Even ultra–low Na^+^ doping is effective in enhancing the chemical interaction between B–site and X–site ions and helps suppress the formation of halogen vacancy defects [21]. The lithium cation (Li^+^) in the perovskites weakens the electron–phonon coupling and acts as a donor in perovskites, suppressing the photo–induced trapping and non–radiative recombination of carriers in perovskites [22]. However, the lattice distortion induced by the inclusion of alkali metal elements in perovskites has been rarely discussed.

In the process of photo–excitation of lead halide perovskite nanocrystals, the photo–excited hot carriers will redistribute energy and relax via various pathways to reach thermal equilibrium with the lattice [23]. Excitation creates free carriers, which are the so–called “hot carrier” in perovskite [24]. Hot carriers will thermalize among themselves through carrier–carrier interactions and scattering, which is known as “carrier thermalization” [25]. Then, the carriers will equilibrate with the lattice, mainly through inelastic electron–phonon coupling, and will pass energy to the lattice, becoming “cold”, a phenomenon known as “carrier cooling” [26]. The electron–phonon coupling is one of the most fundamental ultrafast processes, which plays an important role in the efficiency of energy conversion of perovskite [27]. However, the origins of the mechanisms behind the carrier cooling and the methods of tuning electron–phonon coupling in perovskite remain unclear [28].

In this work, the effect of the addition of alkali metal cation Cs^+^ in the precursors for the synthesis of perovskite CsPbBr_3_ nanocrystals is systematically studied. It is found that the addition of alkali metal cation Cs^+^ in precursors reduces lattice strain in perovskites and results in the emergence of a new phase that coexists with the cubic phase of CsPbBr_3_. In particular, upon light illumination, the lattice strain will be compensated by the light–induced lattice expansion, significantly reducing the electron–phonon coupling, thus slowing down the hot–carrier cooling in perovskites. Our finding provides a strategy for tuning the electron–phonon coupling and carrier cooling in perovskites.

## 2. Materials and Methods

### 2.1. Materials

Lead bromide (PbBr_2_, Aladdin, 99.0%, AR), cesium bromide (CsBr, Aladdin, 99.9%, metals basis), oleic acid (OA, Aladdin, 99.0%, GC), N,N–dimethylformamide (DMF, Aladdin, 99.5%, AR), dimethyl sulfoxide (DMSO, Aladdin, 99.5%, AR), toluene (Aladdin, ≥99.5%). All chemicals were purchased and used without further purification.

### 2.2. Synthesis

The perovskite nanocrystal samples used in this work are synthesized using a typical supersaturated recrystallization synthesis process at room temperature. CsBr and PbBr_2_ are solved in DMF, DMSO, or DMF/DMSO mixture solutions and are loaded into a 50 mL beaker and stirred vigorously by magnetic stirrers. The samples S1–S5 can be obtained under stoichiometric and nonstoichiometric conditions by adjusting the amount of CsBr. After about 4 h, the powder of CsBr and PbBr_2_ is completely solved. Then, an appropriate amount of OA (typically 1 mL) is added to the solution, followed by continuous stirring for 1 h, until the solution is well mixed, colorless, transparent, and clear. Then, 1 mL of the precursor solution is quickly added into 10 mL toluene with vigorous stirring. After reaction for 60 s, the solution is transferred to a sealed glass bottle. (See Table 1).

### 2.3. Characterization

The samples were measured by X-ray diffraction (XRD) Rigaku Smartlab 9 kW. The X-ray source includes a tungsten (W) cathode and copper (Cu) anode, which work on 9 kW and have the Kα wavelength of Cu. The X-ray photoelectron spectroscopy (XPS) characterizations were measured by PHI 5000 Versaprobe III. The X-ray source has the Kα wavelength of aluminum (Al). A photoluminescence (PL) spectra was obtained by Princeton Instrument’s (PI) SP2300 with the CCD detector PIX400BRX under excited 325 nm CW He–Cd laser. Sample cells were cooled by the optical cryostat (JANIS SHI–4), which could operate at a range of 4 K to 325 K. The operation temperature was recorded by a Lakeshore 336 temperature controller to obtain the temperature–dependent PL spectra. The time–resolved PL (TRPL) was measured by a time–correlated single photon counting system of Picoharp–300 Synchronization under excitation of 375 nm pulse laser (40 ps). In the transient absorption spectra (TAS) measurement, the perovskite samples were excited by an ultrafast pulse laser with a wavelength of 355 nm, a pulse width of 100 fs, and a repetition rate of 1 kHz (Coherent Astrella ultrafast Ti:Sapphire laser with OperA Solo). The ultra–continue white light source was obtained through the crystal of CaF2 excited by the same ultrafast pulse laser with a wavelength of 800 nm.

## 3. Results and Discussion

**Figure 1:**
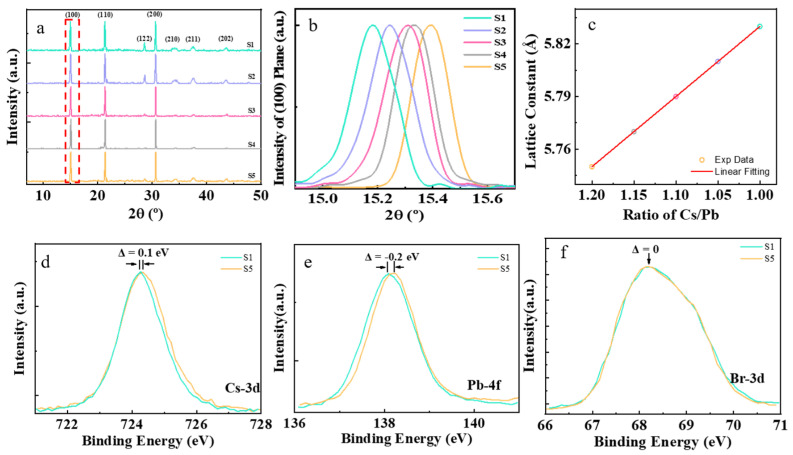
The XRD and XPS results of the perovskite CsPbBr_3_ nanocrystal samples. (**a**) General XRD results of samples S1–S5. (**b**) Plane (100) XRD result of samples S1–S5. (**c**) The relationship of the distance of planes (100) series with a ratio of Cs/Pb in precursors. (**d**–**f**) The binding energy of Cs–3d, Pb–4f, and Br–3d from stoichiometric samples S1 to nonstoichiometric samples S5.

Five samples, S1–S5, were synthesized with different amounts of Cs^+^ cations in the precursors, as shown in Table 1. Figure 1 presents XRD and XPS characterizations of the perovskite CsPbBr_3_ nanocrystal samples S1–S5 that were synthesized. The XRD characterization results of the samples are shown in Figure 1a. There are three peaks in the XRD diffraction patterns, located at about 15°, 21°, and 30°, respectively, which can be assigned to the diffractions of (100), (110), and (200) planes of the cubic phase of perovskite CsPbBr_3_. It is interesting to note that the diffraction peak of (100) plane shifts toward a bigger Bragg angle in order for samples from S1 to S5, as shown in Figure 1b. The interplanar distance of plane (100) can be obtained from the XRD pattern using Bragg’s equation [29]:

(1) $$d_{100}=\frac{n\lambda }{2\sin\theta },$$

where *d* is the (100) interplanar distance, *n* is the order of reflection, *λ* is the wavelength of X-ray used in characterization, and θ is the Bragg angle. The interplanar distance of the (100) plane linearly decreased with the Cs^+^ cations in precursors (ratio of Cs/Pb), evidenced that the Cs^+^ cations were successfully incorporated into the lattice of the perovskite nanocrystals.

The soft nature of lead halide perovskites makes them susceptible to strain, which has recently been recognized as a pivotal factor that influences their optoelectronic property and device stability of perovskites. Strain refers to the relative deformation of a crystal structure due to external stress and other factors. It can be described by Williamson–Hall’s equation [30]:

(2) $$\varepsilon =\frac{a_{0}-a}{a_{0}},$$

where ε is the lattice strain, and $a_{0}$ and a are the lattice constants for unstrained and strained lattices, respectively. It is interesting to note that the lattice constant of our samples shifts toward smaller value in the order from S1 to S5, as shown in Figure 1c, suggesting that the existence of lattice strain in nonstoichiometric samples S2–S5, compared to the stoichiometric sample S1. The role of the alkali metal cations has usually been suggested to be the passivation of the defects at the surface and grain boundaries of perovskite nanocrystals [31]. Moreover, the diffraction peaks of plane (200) can be fitted by cubic phase (ICSD No. 054–0752) and monoclinic phase (ICSD No. 054–0751) of perovskite CsPbBr_3_, as shown in Figure S1. The stoichiometric sample S1 was mainly composed of the cubic phase CsPbBr_3_. After adding excessive Cs^+^ cations in the precursors, there were more monoclinic phases coexisting in nonstoichiometric samples S2–S5. The monoclinic phase of CsPbBr_3_ has a larger cell volume than the cubic phase, resulting in lattice strain in the nonstoichiometric samples S2–S5.

To further confirm the relationship between the lattice strain in perovskite CsPbBr_3_ nanocrystals and the excessive addition of the Cs^+^ cations in precursors, the XPS characterizations were conducted on stoichiometric sample S1 and nonstoichiometric sample S5. It was found that the binding energy of Cs–3d in the nonstoichiometric sample S5 had a shift of 0.1 eV toward higher energy, compared with the stoichiometric sample S1, as shown in Figure 1d. The binding energy of Pb–4f in sample S5 had a shift of 0.2 eV toward lower energy, compared with sample S1, as shown in Figure 1e. However, the binding energy of Br–3d had the same value for the two samples, S1 and S5, as shown in Figure 1f. The broad peak of Br–3d XPS spectra can be decomposed into two peaks centered at 68 eV and 69 eV, respectively, as shown in Figure S2a. They correspond to the ionic bond and covalent bond of Br–Pb, respectively [32]. The sample S1 mainly consists of cubic phase perovskite CsPbBr_3_ nanocrystals, which has been synthesized in stoichiometric condition. There is often halogen deficiency in perovskite nanocrystals [33], which induces trap–sites. The halogen deficiency in perovskite needs to be charge–balanced by A–site cations [34]. The Cs^+^ cations taking the position of halogen deficiency will lead to the coexistence of a monoclinic phase with a cubic phase of CsPbBr_3_. Increasing the Cs^+^ cations in precursors will induce more monoclinic phases emerging in the nanocrystals, resulting in a larger lattice strain due to the structure and lattice mismatch of the two phases. Therefore, the lattice strain shows a linear relationship with the Cs^+^ cations in the precursors, increasing from sample S1 to S5.

**Figure 2:**
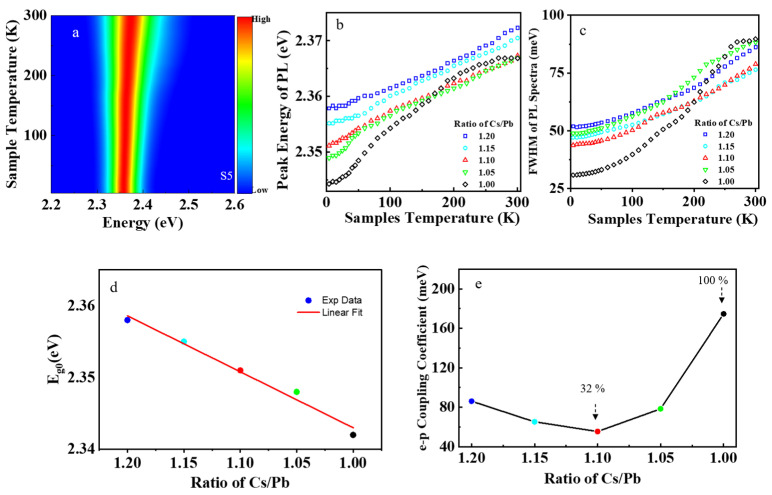
PL spectra of the perovskite CsPbBr_3_ samples. (**a**) The PL spectra mapping of sample S5. (**b**) The peak energy of PL dependent on sample temperature. (**c**) The FWHM of PL spectra dependent on sample temperature. (**d**) The relationship of $E_{g0}$ with a ratio of Cs/Pb in precursors. (**e**) The relationship of electron–phonon coupling with the concentration of Cs^+^ cations in precursors.

The CsPbBr_3_ nanocrystal samples S1–S5 had green emission at room temperature; their PL spectra are shown in Figure 2a and Figure S4. With temperature increase, the PL peak shifted toward higher energy, as shown in Figure 2b. This is because the bandgap of perovskite CsPbBr_3_ increases with sample temperature, which is contrary to most semiconductors. Including the effect of thermal expansion and electron–phonon coupling, the temperature–dependent PL peak energy of perovskite nanocrystals can be described by the equation [35]:

(3) $$E_{g}\left(T\right)=E_{g0}+\alpha \cdot T-\gamma_{LO}\cdot \left(1+\frac{2}{e^{\frac{E_{P}}{k_{B}T}}-1}\right),$$

where $E_{g}\left(T\right)$ is the PL bandgap energy at temperature T, $E_{g0}$ is the bandgap energy at absolute temperature T = 0, α·T is the thermal expansion term, $E_{P}$ is the optical phonon energy, $\gamma_{LO}$ is the electron–phonon coupling coefficient, and $k_{B}$ is the Boltzmann constant. The experimental results show that $E_{g0}$ has a linear relationship with the Cs^+^ cations in precursors, as shown in Figure 2d. The incorporation of excessive Cs^+^ cations in the lattice leads to the emergence of monoclinic phase CsPbBr_3_ in these nanocrystal samples. In stoichiometric perovskite, the 3D frame network of vertex–sharing octahedrons determines the electronic structure, especially at the band edge [36]. The monoclinic phase of perovskite CsPbBr_3_ has a larger cell volume than the cubic phase, resulting in lattice strain in nonstoichiometric samples. The lattice strain in the samples leads to the increasing bandgap of perovskite [37]. The bandgap of perovskite CsPbBr_3_ primarily depends on the Pb atom at the B–site and Br atom at the X–site, while the Cs atom at the A–site mainly contributes to the stabilization of the structure, relatively affecting the bandgap less [38]. The valence band maximum of perovskite CsPbBr_3_ is a hybrid bonding state of the 6 s orbital of the Pb atom and the 4p orbital of the Br atom. The conduction band minimum is a hybrid of the 4p orbital of the Pb atom and the 4p orbital of the Br atom with less antibonding and more nonbonding characters, as shown in Figure S5. In the monoclinic phase of perovskite CsPbBr_3_, the neighboring elongated octahedrons are tilted with an angle of less than 180°, which reduces the overlap between the orbitals of the Pb atom and Br atom, compared to the cubic phase of perovskite CsPbBr_3_. Hence, the conduction band minimum will shift by a smaller amount than the valence band maximum upon introducing a structural change, resulting in the increase of the intrinsic bandgap $E_{g0}$ of perovskite with the excess of Cs^+^ cations in the precursors.

The full width at half maximum (FWHM) of the PL spectrum is usually affected by the electron–phonon coupling in a wide range of semiconductors. The temperature–dependent FWHM of the PL spectra can be described by the following equation [27]:

(4) $$\Gamma_{\mathrm{ex}}\left(T\right)=\Gamma_{0}+\sigma \cdot T+\zeta_{LO}\cdot {\left(e^{\frac{E_{P}}{k_{B}T}}-1\right)}^{-1},$$

where $\Gamma_{ex}\left(T\right)$ is the FWHM of the PL spectrum at temperature T, $\Gamma_{0}$ is the inhomogeneous broadening term, σ·T is the homogeneous term, $E_{P}$ is the optical phonon energy, $\zeta_{LO}$ is the electron–phonon coupling coefficient, and $k_{B}$ is the Boltzmann constant. The electron–phonon coupling coefficient $\zeta_{LO}$ can be obtained by fitting the experimental data with Equation (4). It was found that the electron–phonon coupling coefficient first decreased with the Cs^+^ cations (ratio of Cs/Pb), then increased rapidly, as shown in Figure 2e. When the Cs^+^ cations were 10% excessive, the electron–phonon coupling coefficient $\zeta_{LO}$ reached the minimum value in sample S3, decreased by 32% compared to that of the stoichiometric sample S1.

**Figure 3:**
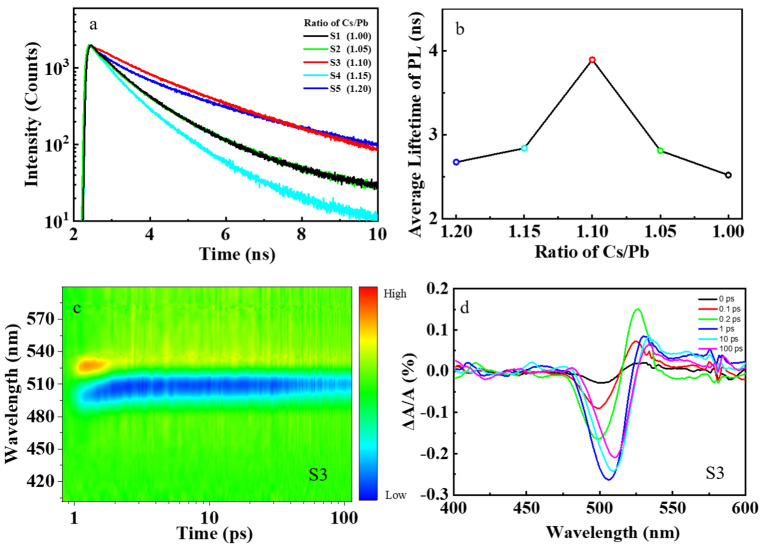
PL decay and TAS results of perovskite CsPbBr_3_ samples. (**a**) The PL decay of samples S1–S5. (**b**) The relationship between average PL lifetime and concentration of Cs^+^ cations in precursors. (**c**) The TAS result of nonstoichiometric sample S3. (**d**) Several representative TAS of nonstoichiometric sample S3.

The electron–phonon coupling will also affect the carrier dynamics. To investigate the dynamics of carrier recombination in our perovskite nanocrystal samples, the TRPL measurements of samples S1–S5 were carried out, and the PL decay traces are shown in Figure 3a. The radiative recombination of photocarriers consists of direct and indirect recombination processes. These two recombination processes can be disentangled because their characteristic times differ significantly from each other [39]. The PL decay trances can be fitted with a bi–exponential decay function to obtain the average PL lifetime $\tau_{ave}$ [40]:

(5) $$I\left(t\right)e^{\frac{-t}{\tau_{ave}}}=I_{1}\left(t\right)e^{\frac{-t}{\tau_{1}}}+I_{2}\left(t\right)e^{\frac{-t}{\tau_{2}}},$$

(6) $$\tau_{ave}=\frac{I_{1}\tau_{1}^{2}+I_{2}\tau_{2}^{2}}{I_{1}\tau_{1}+I_{2}\tau_{2}},$$

where $I\left(t\right)$ is the PL intensity, $I_{1}\left(t\right)$ is the intensity of PL due to direct recombination of photocarriers, $I_{2}\left(t\right)$ is the intensity of PL due to the indirect recombination of photocarriers, $\tau_{1}$ and $\tau_{2}$ are the characteristic lifetimes of direct and indirect recombination of photocarriers, respectively. The nonstoichiometric sample S3 had the longest average lifetime compared to other samples, as shown in Figure 3b. After photoexcitation, electrons will transit to the conduction band and then relax to the band edge, recombine with holes to generate photoluminescence. We call this process the direct recombination process. The photocarriers can also be trapped by defects and then detraped back to the band edge to recombine with holes in the valence band, generating photoluminescence. We call this process the indirect photoluminescence process. This process usually takes a longer time since it involves carrier trapping, de–trapping, and recombination. In nonstoichiometric sample S3, the indirect photoluminescence process plays a more important role, explaining why sample S3 has a longer PL lifetime compared with other samples [41].

**Figure 4:**
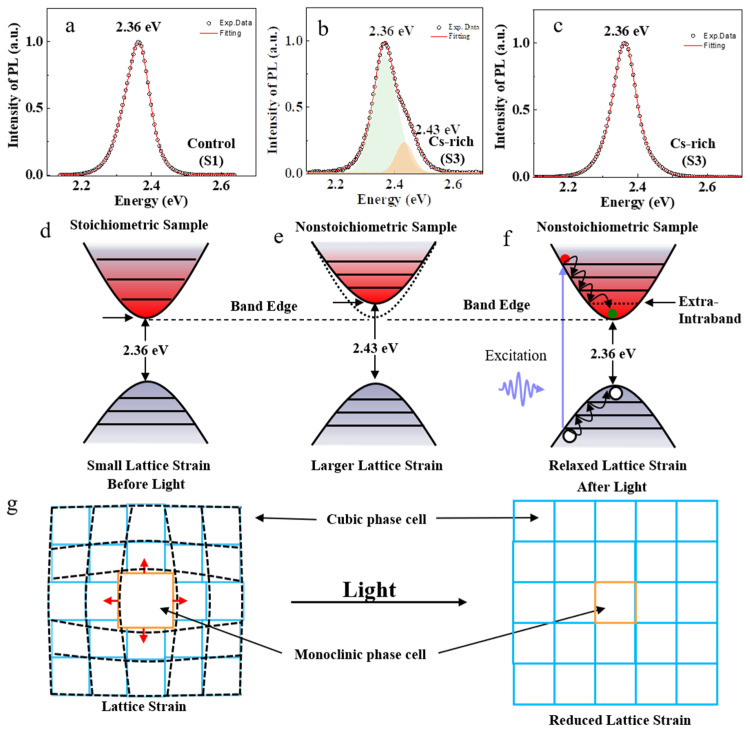
Schematic illustration of the lattice strain and compensation of light–induced lattice expansion in perovskite nanocrystal. (**a**) PL spectrum of stoichiometric sample S1. (**b**) PL spectrum of nonstoichiometric sample S3 excited by low power. (**c**) PL spectrum of nonstoichiometric sample S3 excited by high power. There is obviously a higher energy peak (2.43 eV) absent, which can be explained by the light–induced lattice expansion. (**d**) Schematic illustration of the bandgap of stoichiometric sample. (**e**) Schematic illustration of the bandgap of nonstoichiometric sample. (**f**) Schematic illustration of extra–intra–band level in the bandgap of the stoichiometric sample by light–induced lattice expansion. (**g**) Light–induced lattice expansion reduced lattice strain in perovskite nanocrystal.

Therefore, cubic phase perovskite CsPbBr_3_ nanocrystals are usually formed under stoichiometric conditions. The PL peak energy of the stoichiometric sample S1 was about 2.36 eV, as shown in Figure 4a,d. Our experimental results show that there were two PL peaks in the nonstoichiometric sample S3 under lower power laser excitation, including the lower energy PL at about 2.36 eV and the higher energy one at about 2.43 eV, as shown in Figure 4b,e. The lower energy PL peak of 2.36 eV can be attributed to the cubic phase of perovskite CsPbBr_3_, while the higher energy PL peak of 2.43 eV is due to the lattice strain.

Upon high–power light illumination, the electrons transit from the Pb–6s–Br–4p orbitals to the Pb–6p orbitals, forming weakly bound excitons that are easily dissociated by thermal energy. The electronic transition directly leads to the reduction of electron density at the Br site, which straightens the Pb–Br–Pb bond and results in a larger interatomic spacing. This causes lattice expansion and reduces the local lattice strain [42], as shown in Figure 4g. The experimental PL results of nonstoichiometric sample S3 excited by higher power are shown in Figure 4c. There is only one PL peak of about 2.36 eV, and the higher energy PL peak of about 2.43 eV disappears.

The light–induced lattice expansion can also be interpreted as a consequence of the converse piezoelectric effect [43]. Perovskites are polar materials because of the displacement of the Pb^2+^ cation from the center of the octahedron [44]. In polar materials, the photogenerated carriers are spontaneously separated to produce an effective electric field that deforms the lattice through the piezoelectric tensor. Additionally, the lattice expansion may occur from less–distorted Pb–Br–Pb bonds [45] and tilted adjacent octahedrons [46].

**Figure 5:**
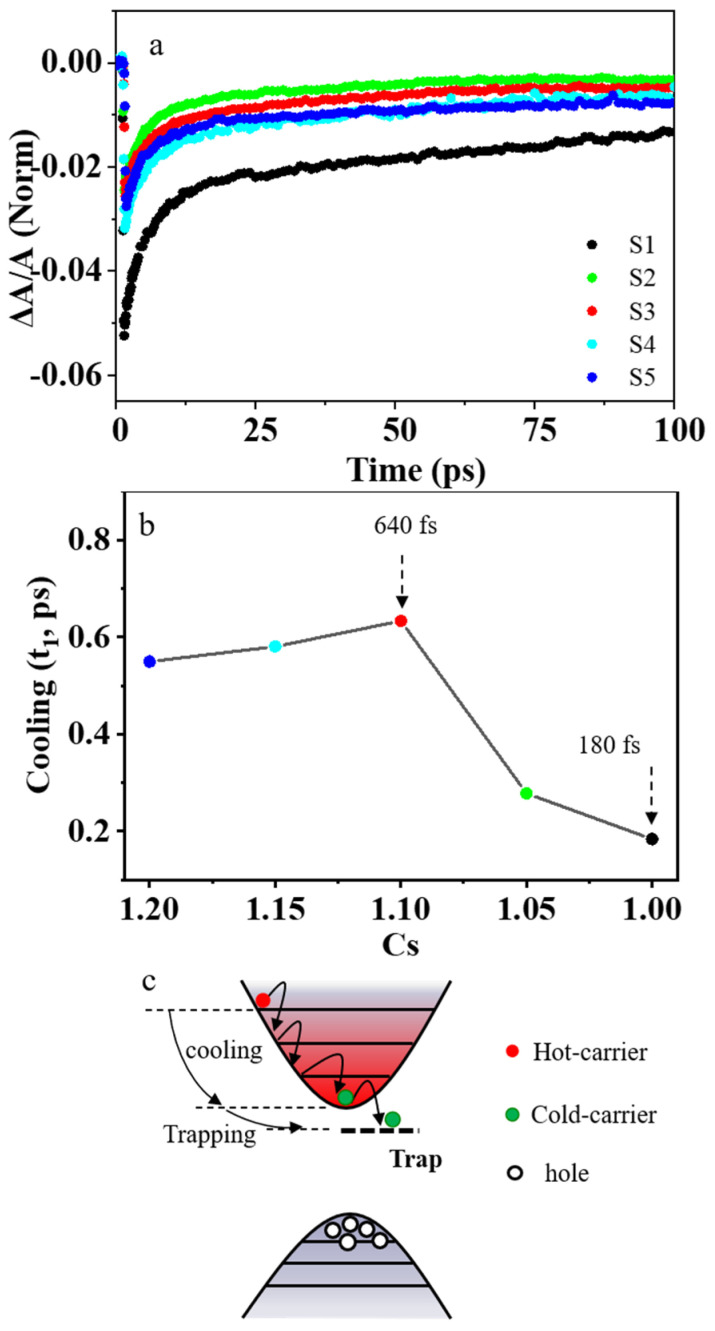
Cooling of carriers in perovskite CsPbBr_3_ samples. (**a**) The PB decay of samples S1–S5. (**b**) The relationship between hot–carrier cooling and concentration of Cs^+^ cations in precursors. (**c**) Schematic illustration of hot–carrier relaxation.

To further explore the ultrafast charge transfer process in perovskite CsPbBr_3_ nanocrystal, we carry out TAS characterization for samples S1–S5, as shown in Figure 3c and Figure S6. The TAS for the sample S3 at several representative delays from 0 to 100 ps are shown in Figure 3d. There is a negative probe bleach (PB) profile, followed by a positive photo–induced absorption (PIA) profile [47]. We can attribute the PIA profile to the photo–induced absorption from the lowest excitonic state to the higher–lying excitonic state in CsPbBr_3_ nanocrystals, and the PB is due to the band–edge bleaching effect [48]. The PB occurs upon excitation by the pump pulse because the electrons in the conduction band and the holes in the valence band can reduce the absorption of probe photons [49]. When the CsPbBr_3_ nanocrystals are excited into the higher excitonic state, TAS is dominated by the intense PIA during the initial delay times. As electrons accumulate in the lowest–energy states in time, the PIA signal eventually decays and is replaced by a strong band–edge bleaching due to state filling [50]. Our experimental results show that the normalized PB of nonstoichiometric sample S3 decays more slowly compared with the stoichiometric sample S1, as shown in Figure 5a. The PB decays in these perovskite samples S1–S5 can be fitted with a bi–exponential decay function of Equation (5) to obtain the hot–carrier cooling time. The stoichiometric perovskite sample S1 has a very short hot–carrier cooling time of about 180 fs. However, the nonstoichiometric perovskite sample S3 has a much longer hot–carrier cooling of about 640 fs, which is about 3.5 times longer than that of sample S1, as shown in Figure 5b. The longer hot–carrier cooling time is due to the smaller electron–phonon coupling coefficient in sample S3. After photoexcitation, the hot carriers will relax to the band edge. As shown in Figure 5c, the hot carriers usually lose energy to the optical phonons (mainly LO phonons). The LO phonons decay into acoustic phonons, and the energy then dissipates into the lattice as heat [23].

**Figure 6:**
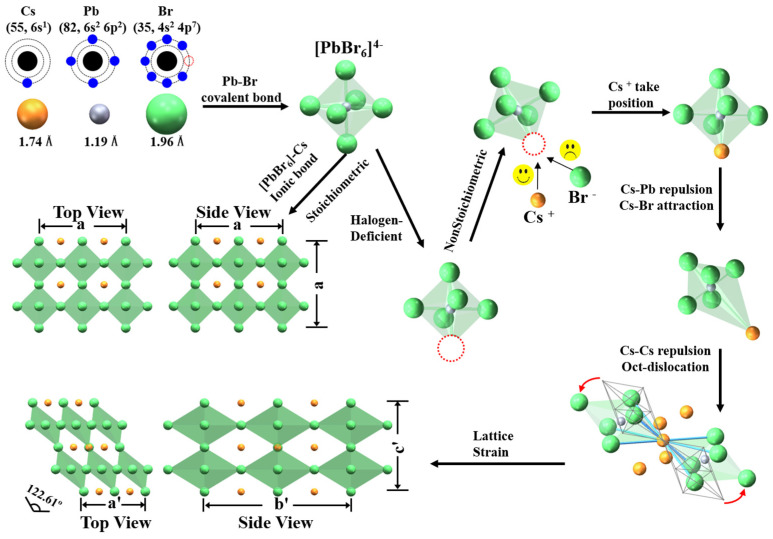
Schematic illustration of the crystallization kinetics via rationale adjustment of the concentration of alkaline cation.

To analyze how the excess Cs^+^ cations in precursors influence the lattice of perovskite, we sketched the schematic of the solution synthesis process of perovskite nanocrystal in Figure 6. The positively charged Pb^2+^ cation has heavier mass, more charges, and a smaller ionic radius than the positively charged Cs^+^ cation. It is easier to form the covalent bond of Pb–Br than the ionic bond of Cs–Br. At the beginning of seed–crystal growth, one Pb^2+^ cation coordinates with 6 Br^−^ anions, forming a stable cage of regular octahedron [PbBr_6_]^4−^. Then, one negatively charged octahedron [PbBr_6_]^4−^ coordinates with eight Cs^+^ cations in a three–dimensional (3D) frame network. In the 3D frame network, each octahedron shares a vertex with the nearest neighboring octahedron. Cs^+^ cations satisfy the requirements of stabilizing steric structure and charge balance.

However, the solution–based synthesis of perovskite nanocrystals invariably introduces defects in the samples, including halogen deficiency [51]. Under nonstoichiometric conditions, the negatively charged octahedrons [PbBr_6_]^4−^ can coordinate with positively charged excessive Cs^+^ cations because of Coulomb interaction. There is repulsion between positively charged Cs^+^ and positively charged Pb^2+^ cations; the latter takes the center site of the octahedron. There is attraction between the positively charged Cs^+^ cation and negatively charged Br^−^ anion, which takes the vertex sites of octahedrons. When the excessive Cs^+^ cations take the sites of halogen deficiency, the regular octahedrons will deform into elongated octahedrons because of the charge rebalance in space. Therefore, there is a stretch of the covalent Pb–Br bond and compression of the ionic Cs–Br bond in octahedrons, forming elongated octahedrons. The neighboring elongated octahedrons are not stable in the 3D frame network, and tilt around the sharing vertex, promoting the formation of monoclinic phase perovskite CsPbBr_3_. Therefore, there is a monoclinic phase coexisting with cubic phase in perovskite CsPbBr_3_ nanocrystals under the nonstoichiometric condition. The monoclinic phase has a larger cell volume than the cubic phase, resulting in structure mismatch and lattice strain in nonstoichiometric samples.

## 4. Conclusions

In summary, how the excessive Cs^+^ cations influence the lattice of perovskite CsPbBr_3_ nanocrystals has been investigated in this work. It has been discovered that there is lattice strain in perovskite CsPbBr_3_ nanocrystal samples synthesized under nonstoichiometric conditions. A new monoclinic phase coexists with the cubic phase in perovskite CsPbBr_3_ nanocrystals. When the Cs^+^ cations have an excessive value of about 10% in precursors, The electron–phonon coupling decreases by about 70%, and the cooling time of hot–carriers increases by about 3.5 times, which can be explained in terms of lattice strain in nonstoichiometric samples. The lattice strain can be compensated by light–induced lattice expansion when the sample is photoexcited. This work will provide a new way to improve the properties of perovskite materials.

## Figures and Tables

**Table 1:** The amount of chemicals used in the synthesis of perovskite CsPbBr_3_ nanocrystals.

Samples	S1	S2	S3	S4	S5
CsBr (mg)	85.10	89.36	93.61	97.87	102.12
PbBr_2_ (mg)	146.70	146.70	146.70	146.70	146.70
Cs:Pb	1.00:1	1.05:1	1.10:1	1.15:1	1.20:1

## Data Availability

Data available upon written request to the corresponding author.

## Supplementary Materials

The following supporting information can be downloaded at: https://www.mdpi.com/article/10.3390/nano13243134/s1, Figure S1: The XRD results of perovskite nanocrystal samples; Figure S2: the XPS results of perovskite nanocrystal samples; Figure S3: Lattice of perovskite CsPbBr_3_; Figure S4: Temperature–dependent PL spectra of perovskite CsPbBr_3_ samples; Figure S5: Schematic illustration of lattice, conduction band, and valence band of perovskite CsPbBr_3_; Figure S6: The TAS results of perovskite CsPbBr_3_ samples; Figure S7: The SEM results of perovskite CsPbBr_3_ samples.
