# Supplementary material for: A Strategy for Tuning Electron–Phonon Coupling and Carrier Cooling in Lead Halide Perovskite Nanocrystals
Source: Nanomaterials (Basel). 2023 Dec 13;13(24):3134. doi: 10.3390/nano13243134 (PMC10745929; doi:10.3390/nano13243134)
Supplement: Supplementary file 1 [file nanomaterials-13-03134-s001.zip › nanomaterials-2745246-Supplementary.pdf]

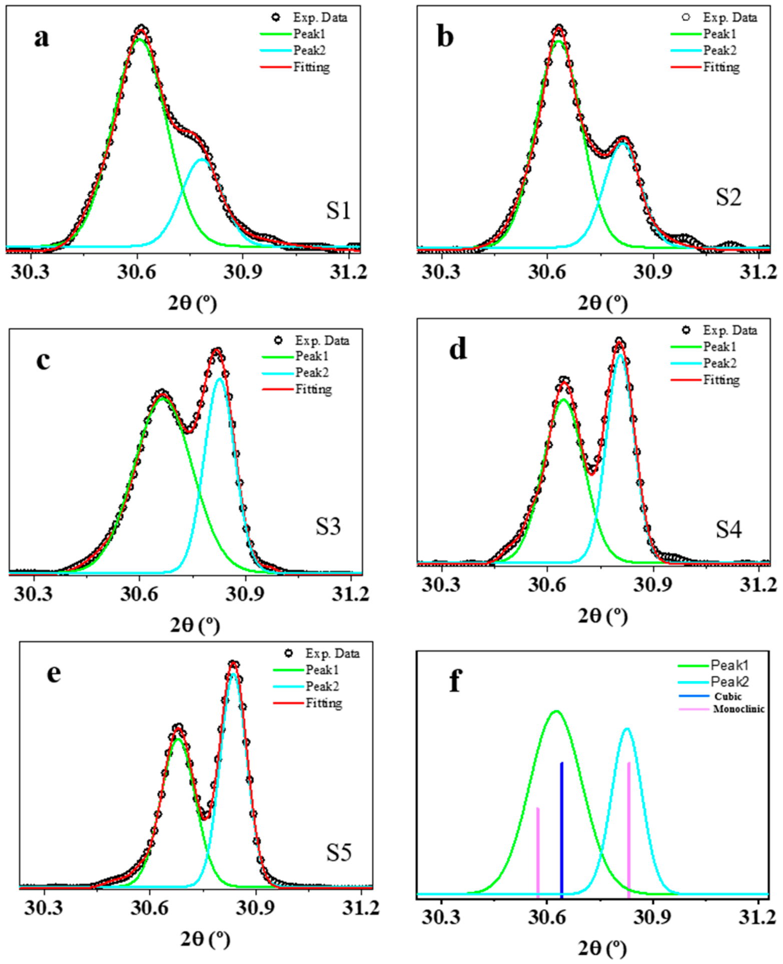

Figure S1. XRD results of perovskite nanocrystal samples. (**a-e**) perovskite sample S1-S5 fitted by cubic and monoclinic phase of perovskite $CsPbBr_3$. (**f**) Cubic and monoclinic phase of perovskite $CsPbBr_3$.

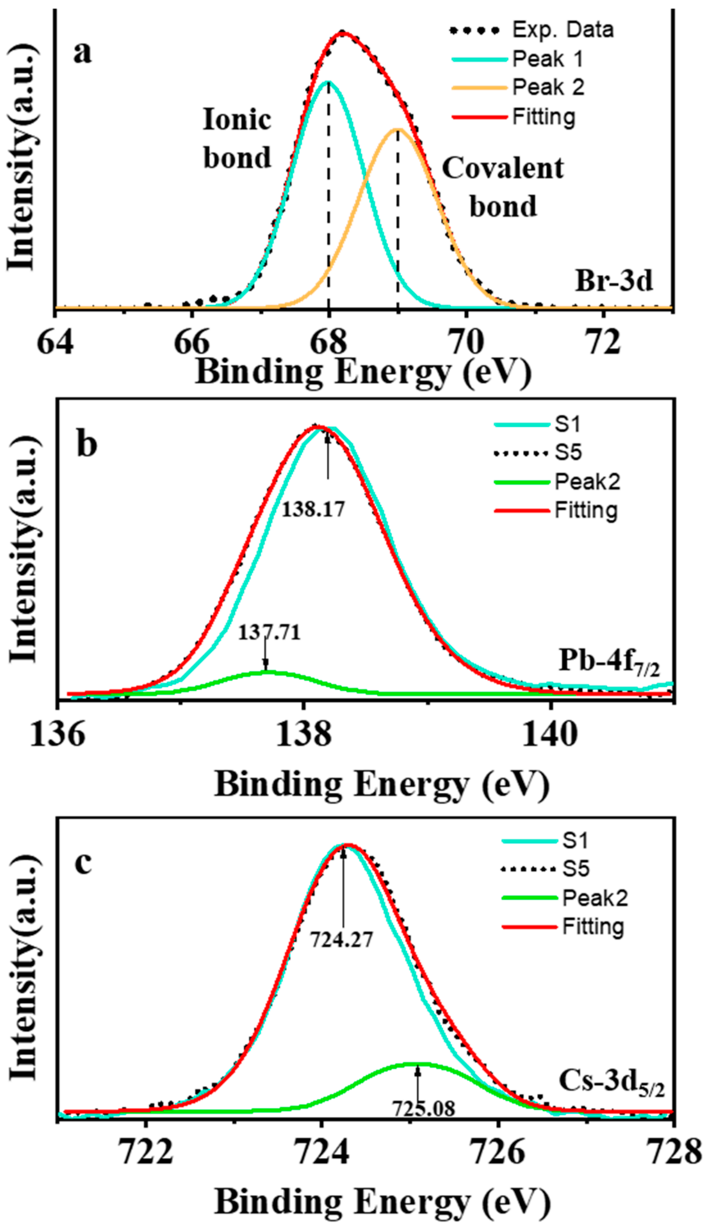

Figure S2. XPS results of perovskite nanocrystal samples. (**a**) The binding energy of Br-3d in perovskite $CsPbBr_3$ samples. (**b**) The binding energy of Pb-$4f_{7/2}$ in stoichiometric sample S1 and nonstoichiometric sample S5. (**c**) The binding energy of Cs-$3d_{5/2}$ in stoichiometric sample S1 and nonstoichiometric sample S5. There is a new peak of 725.08 eV at the higher energy shoulder of the Cs-3d curve in nonstoichiometric sample S5, indicating that the excessive Cs^+ cation have been successful incorporated into the perovskite $CsPbBr_3$ nanocrystals, and coordination with the neighboring atom in the lattice.

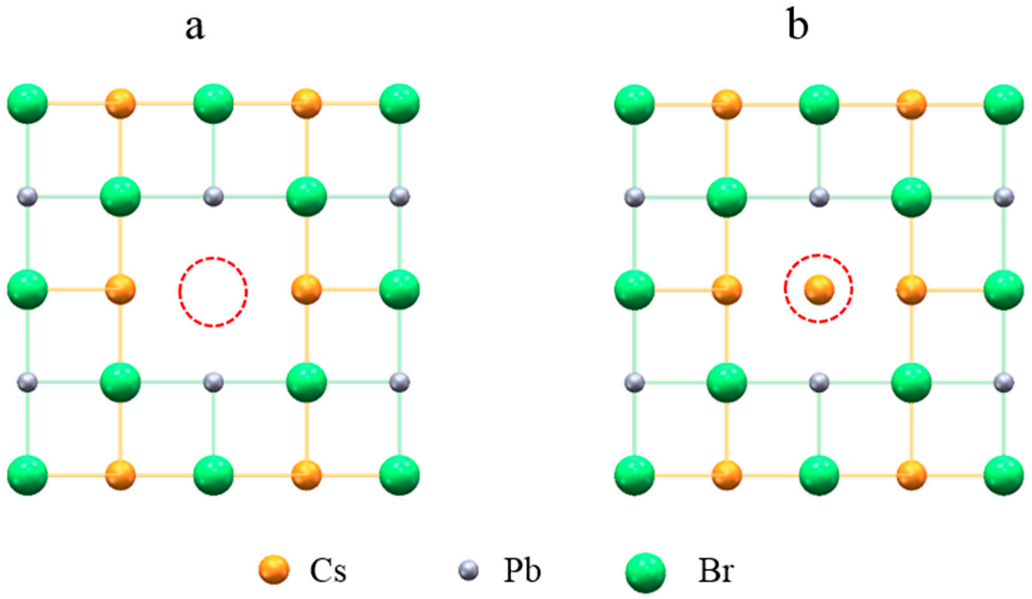

Figure S3. Lattice of perovskite $CsPbBr_3$. (**a**) Haligon-deficient in lattice. (**b**) Cs^+ cation taking the position of haligon-deficient in lattice.

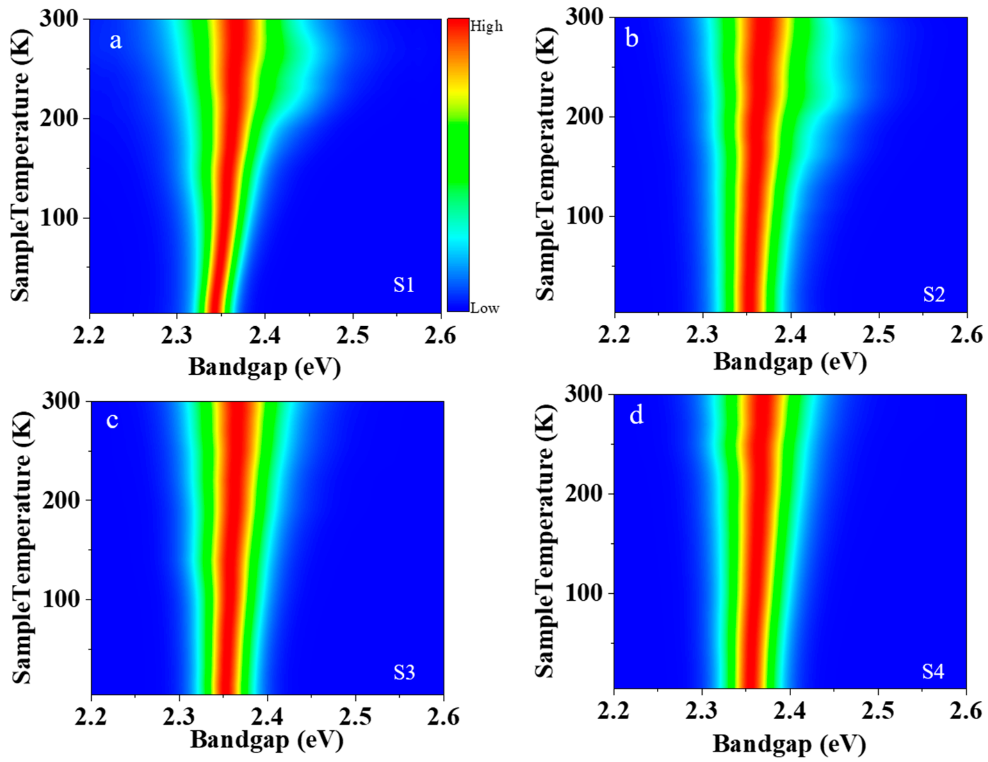

Figure S4. Temperature-dependent PL spectra of perovskite $CsPbBr_3$ samples. (**a**) Temperature-dependent PL spectra of stoichiometric sample S1. (**b**) Temperature-dependent PL spectra of nonstoichiometric sample S2. (**c**) Temperature-dependent PL spectra of nonstoichiometric sample S3. (**d**) Temperature-dependent PL spectra of stoichiometric sample S4.

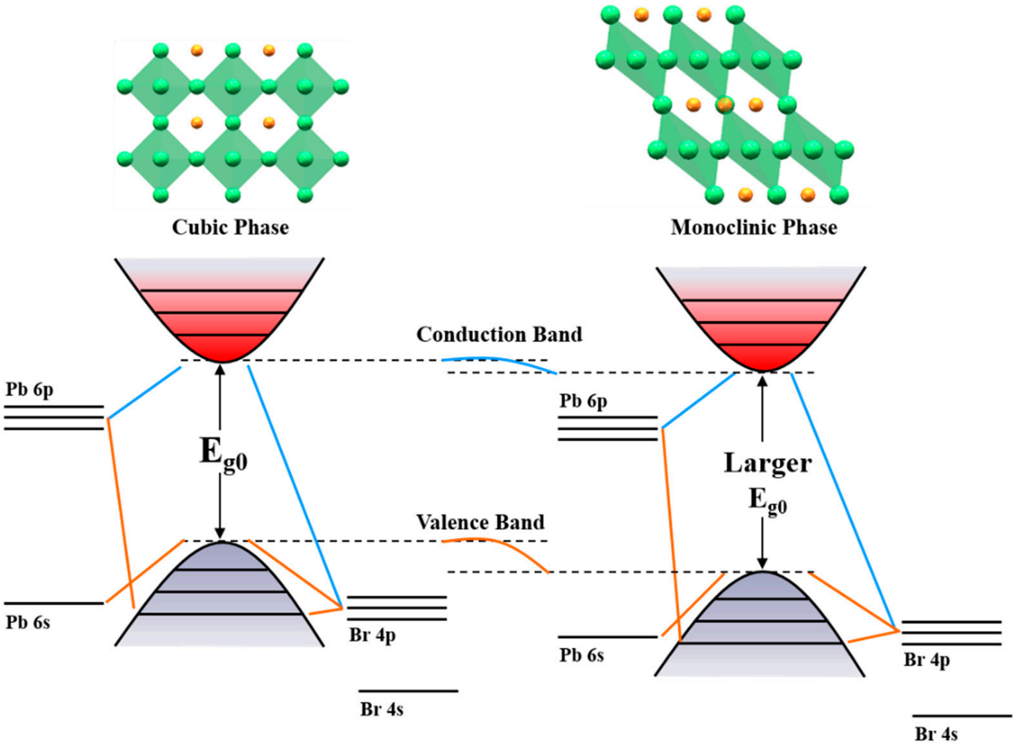

Figure S5. Schematic illustration of lattice, conduction band, and valence band of perovskite $CsPbBr_3$

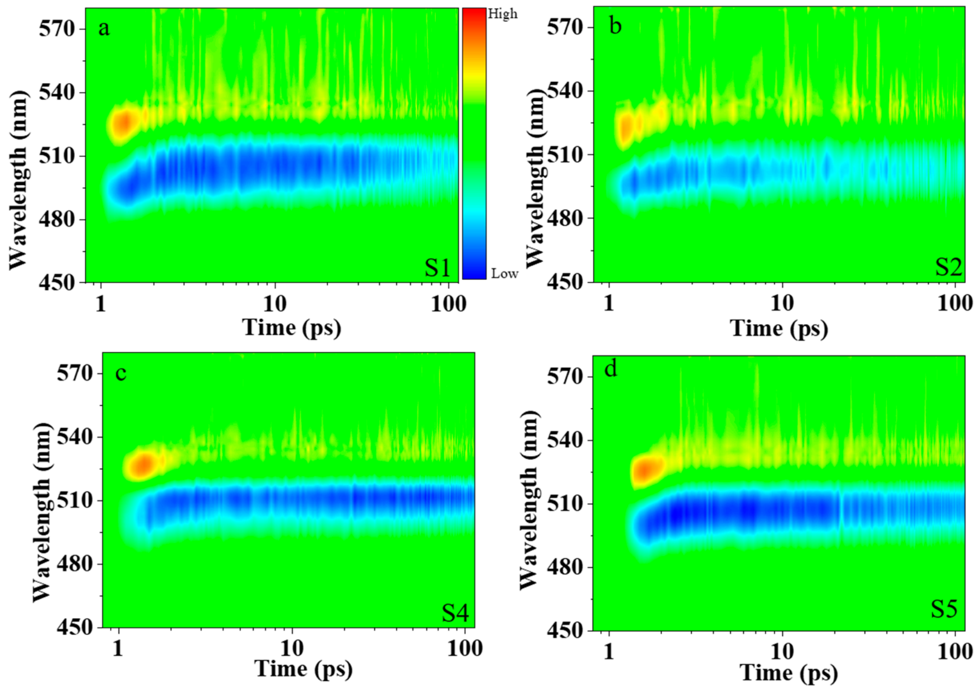

Figure S6. TAS results of perovskite $CsPbBr_3$ samples. (**a**) TAS results of stoichiometric sample S1. (**b**) TAS results of nonstoichiometric sample S2. (**c**) TAS results of nonstoichiometric sample S4. (**d**) TAS results of nonstoichiometric sample S5.

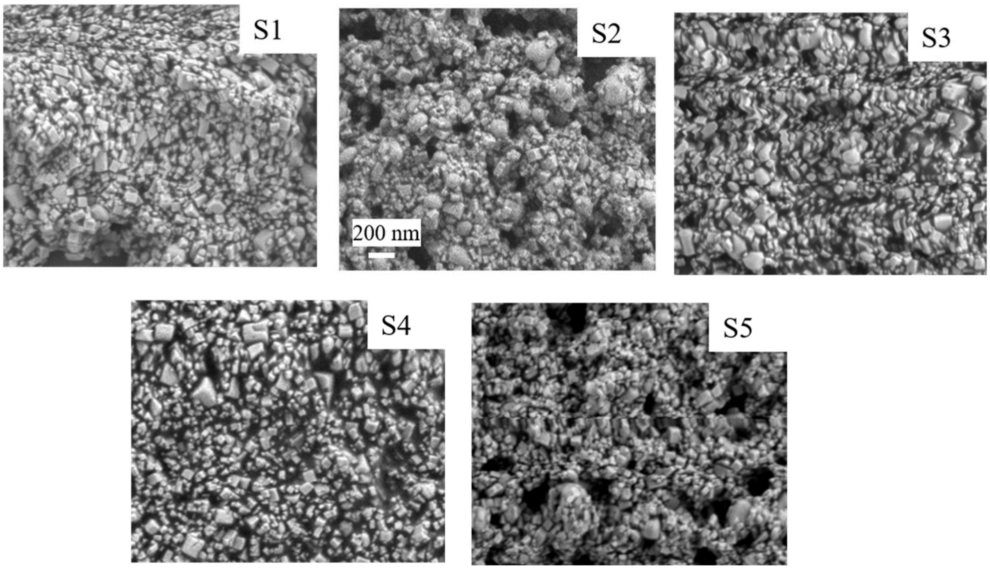

Figure S7. SEM results of perovskite $CsPbBr_3$ samples S1-S5.
